# The influence of lean and fat mass on vascular structure and function in youth of varying body mass index percentiles- a comprehensive approach

**DOI:** 10.3389/fendo.2026.1720148

**Published:** 2026-06-01

**Authors:** Nicholas G. Evanoff, Jack S. Dooley, Aaron S. Kelly, Donald R. Dengel

**Affiliations:** 1 School of Kinesiology, University of Minnesota, Minneapolis, MN, United States; 2 Center for Pediatric Obesity Medicine, Department of Pediatrics, University of Minnesota Medical School, Minneapolis, MN, United States

**Keywords:** abdominal aorta, BMI, body composition, carotid artery, fat mass, lean mass, vascular

## Abstract

**Objective:**

The aim of this study was to compare and evaluate the relationship between body composition and arterial function among youth throughout a range of body mass index (BMI) percentiles.

**Methods:**

Ultrasonographic measurements of the carotid artery and abdominal aorta were obtained from 190 youth (96 female, 12.9 ± 0.2years). Body composition was assessed by dual X-ray absorptiometry. Participants were stratified by BMI as normal weight (NW: <85^th^ percentile; n=79), overweight/obesity (OW/OB: ≥85^th^ to <95^th^ percentile; n=61), and severe obesity (SO: ≥120% of the 95^th^ percentile; n=50). Analysis of variance and linear regression evaluated group differences and the association of body composition variables and arterial function, respectively.

**Results:**

Lean mass (NW = 32.9 ± 9.2kg. OW/OB=25.1 ± 8.8kg, SO = 44.7 ± 12.5kg; p<0.001, all). and fat mass (NW = 10.8 ± 10.5 kg. OW/OB=39.3 ± 10.8kg, SO = 48.7 ± 12.6kg) were all found to be significantly higher (p<0.001, all) across increasing BMI groups. Aortic and carotid intima-media thickness was significantly increased between groups (Aortic: NW = 0.59 ± 0.09mm, OW/OB=0.51 ± 0.17mm, SO = 0.43 ± 0.17mm; Carotid: NW = 0.54 ± 0.05mm, OW/OB=0.51 ± 0.07mm, SO = 0.48mm ± 0.11; p<0.0001 for all groups). Aortic cross-sectional distensibility was significantly (p=0.033) elevated between the SO (30.61 ± 1.48%) and NW (36.39 ± 1.49%) but not observed in the carotid artery. Aortic incremental elastic modulus (IEM) was significantly greater in the SO (1153.0 ± 70.8mmHg) group compared to OW/OB (960.4 ± 48.6mmHg) and NW (846.0 ± 40.9mmHg) groups (p<0.001). Carotid IEM was significantly greater (p=0.005) in SO (1191.07 ± 383.09mmHg) compared NW (998.91 ± 313.19mmHg). LM and LMI were similarly associated with increased compliance in the abdominal aorta, but not the carotid artery. FM and FMI were similarly associated with greater arterial stiffness in both arterial beds with absolute FM showing more consistent associations with decreased vascular function.

**Conclusion:**

Vascular structure and function vary across BMI percentiles in children and adolescents. Lean mass and fat mass relate to both arterial structure and function differently in different vascular locations, especially in the abdominal aorta. Lean mass displays a protective vascular effect in the abdominal aorta more than the carotid while fat mass displays a negative effect in both arterial beds.

## Introduction

Measures of vascular structure and function such as intima media thickness (IMT), compliance, and distensibility are well-established surrogate biomarkers for cardiovascular disease (CVD) risk (1–3). While most of the research exploring this relationship has been done in adults, changes in these vascular measures have also been observed in children and adolescents (4, 5). Similar to adults, obesity in children and adolescent has been linked to alterations in both vascular structure and function (5–7). Clinically, Body Mass Index (BMI) is used in both adults and children to stratify obesity and CVD risk (8). However, less is known about if the physiological (absolute mass) and computational (height adjusted) components within BMI in children are due to early pathological changes or represent adaptations to normal growth and development (9).

Both fat mass (FM) and lean mass (LM) are commonly reported in in the literature as well as within body composition reports. Furthermore, the lean and fat components of BMI (lean mass index [LMI] and fat mass index [FMI]) are being reported more and more in the literature and have been shown to relate differently to CVD risk in adults (10–12). This has resulted in come confusion, clinically, since it is possible that FM and/or LM as well as LMI and/or FMI may relate to vascular health differently in children and adolescents (13, 14).

In a large cohort of 8- to 9-year-olds who had measures of body composition and vascular structure and function, Sletner et al. (14) reported that differences in vascular structure and function were related to BMI and FM. However, for some of the vascular outcomes, the relations with BMI were explained by LM (14). Sletner et al. (14) concluded that, in children, differences in vascular structure and function that are often attributed to BMI may actually represent the adverse effects of FM. In addition, these changes in vascular health may also be due to the adaptive effects of increasing body size due to growth and development as well as the protective effects of increasing LM independent of height (14). Therefore, it’s important to elucidate the contribution of these body composition biomarkers more fully on vascular function in pediatric populations.

Due to its superficial location the common carotid artery is frequently used to assess arterial structure and function. The carotid IMT (cIMT) has become a well-accepted independent predictor of cardiovascular events and disease risk (15–17). Beyond cIMT, functional variables have also been examined mostly in relation to the carotid artery in adults and children (14, 18–20) and have been also shown to be associated with CVD risk (21–23). However, it is possible that other arteries besides the carotid may add important prognostic value for assessing CVD risk (24, 25). In addition, it is not known if all arterial beds in the human body react the same to CVD risk factors.

The abdominal aorta may be one artery that offers new insight into the development of CVD given that it makes the largest contribution to buffering arterial pressure (26). In addition, the abdominal aorta is composed of more elastin than smaller arteries, and may display pathologies differently than the peripheral arteries (i.e. carotid) (27). In one of the few studies that examined the abdominal aorta in children, Harbin et al. (28) found distensibility and elasticity differences in the abdominal aorta in children and adolescents exposed to secondhand smoke. While in that same study (28) there were no changes in carotid structure, and functional differences, due to secondhand smoke. In general, there is a lack of information on body composition biomarkers in relation to arterial health, especially related to the abdominal aorta, in younger populations of varying BMI ranges.

The present study aimed to compare body composition biomarkers (absolute tissue unadjusted for height: LM, FM; tissue relative to height: LMI, FMI) and vascular structural and functional parameters (IMT, compliance, distensibility, incremental elastic modulus), of the carotid and abdominal aorta arteries in children and adolescents of varying BMI percentiles. In addition, we explored the relationship between these body composition biomarkers and vascular structure and function in the common carotid artery and the abdominal aorta. We also examined the independent contributions of body composition biomarkers by cross adjusting models: LM for FM and LMI for FMI. This approach provides a more comprehensive view of their individual contributions. Using this multiple dual approach, pediatric clinicians and researchers may find insight into how best to interpret body composition components relating to CVD risk more appropriately.

## Materials and methods

### Study participants and inclusion/exclusion criteria

This exploratory analysis included one hundred and ninety children and adolescents (92 males) between the ages of 8 and 17 years who participated in a cross-sectional study addressing cardiovascular risk factors and vascular function (29). Participants were stratified by BMI as normal weight (NW: <85^th^ percentile; n=79), overweight/obesity (OW/OB: ≥85^th^ to <120% of the 95^th^ percentile; n=61), and severe obesity (SO: ≥120% of the 95^th^ percentile; n=50). Only those participants who had abdominal aorta ultrasound imaging were included in this analysis. Participants were excluded on the basis of untreated obstructive sleep apnea, genetic obesity, bariatric surgery, antihypertensive medication use, type 1 and 2 diabetes mellitus, history of hypercholesterolemia, chronic kidney disease, Kawasaki disease, autoimmune inflammatory diseases, and congenital heart disease upon enrollment. Study approval oversight was provided by the University of Minnesota Institutional Review Board and conducted per the Declaration of Helsinki guidelines. Prior to study procedures, parents and participants provided signed informed consent and assent, respectively. All participants were instructed to fast for at least 12-hours, limit strenuous exercise 24 hours prior to study visits, and arrive euhydrated.

### Procedures

#### Anthropometrics, body composition, and clinical assessments

Height and weight were measured by the same calibrated digital scale and wall mounted stadiometer for all participants. BMI was calculated as weight in kilograms (kg) divided by height in meters squared (m^2^). Dual X-ray absorptiometry (DXA; iDXA: General Electronic Medical Systems, Madison WI, USA with enCore software version 16.0) was used to measure and analyze body composition, respectively. In the context of DXA, LM represents lean soft tissue which excludes bone mineral content. A licensed medical provider assessed pubertal maturation using Tanner stages (I–V). Seated blood pressure was collected by an automatic sphygmomanometer after ten-minutes rest in triplicate with 3 minutes between each measurement. The last two systolic and diastolic readings were averaged.

#### Carotid and abdominal aorta ultrasound imaging and analysis

The abdominal aorta was imaged via conventional B-mode ultrasound (Acuson, Sequoia 512, Siemens Medical Solutions USA Inc., Mountain View, CA, USA) using an 8.0-1.0 MHz linear array transducer. The abdominal aorta imaging region of interest was distal to the renal and proximal to the iliac bifurcation. The ultrasound transducer was tilted to achieve an ionization angle of less than 30-degrees with both near and far walls well represented. The carotid artery was imaged sequentially to the abdominal aorta approximately one cm proximal to the carotid bulb. Ten-second cine clips were recorded at 20 frames per second, digitized and sent to a secure computer for later analysis using wall tracking software (Vascular Research Tools 6, Medical Imaging Application, LLC, Iowa City, IA, USA). The diameters were averaged over the recording period for all vascular functional parameters (compliance, distensibility, and incremental elastic modulus). Arterial diameter refers to the end diastolic (maximal) diameter. Functional indices are defined as for both the abdominal aorta and carotid artery (denoted as a = abdominal aorta and c = carotid henceforth) as previously described (18).

### Statistical analysis

All statistics performed in RStudio (version 4.0.1, R Foundation for Statistical Computing, Vienna, Austria). Normality was determined via density plot visualization and the Kolmogorov-Smirnov Normality Test. Descriptive statistics split between BMI percentiles and summarized by mean (SD) or n (%) for continuous and categorical variables, respectively. Analysis of variance (ANOVA) and Bonferroni *post-hoc* pairwise comparison used to determine differences between BMI percentile groups and measures of arterial structure and function. Chi-squared tests were used to the association between categorical variables.

Multiple linear regression was employed to test the associations of LM, FM as well as LMI and FMI with arterial (abdominal aorta and carotid artery) elasticity while controlling for Tanner stage (maturity), race, and sex due to being possible confounders. Tanner stage was included instead of age due to collinearity and Tanner stage’s uneven distribution within our sample. Models were created for absolute tissue (LM, FM) and relative tissue (LMI, FMI) alone and combined in order to represent both *in vivo* conditions as well as mutually adjusted to explore individual effects of the absolute and relative tissues.

Additionally, to account for multiple comparisons across the 14 tests within each model, p-values were adjusted using the Benjamini-Hochberg (BH) procedure to control the False Discovery Rate (FDR) at the 0.05 level. This approach was selected to balance the risk of Type I errors against the need for sufficient statistical power in this exploratory analysis (30). Interaction terms were found insignificant and, therefore, excluded from the final models. A two-sided alpha level of 0.05 denotes statistical significance.

## Results

### Participant attributes

Descriptive characteristics of participants are shown in **Table 1**. There were no significant differences in age and sex between the different BMI stratified groups. The participants were primarily white, and race was proportional across groups. There were no significant differences in pubertal status. However, the NW participants were skewed toward Tanner stage 1 (35.5%). The SO were significantly taller, on average, than the NW (*p* = 0.009) while the OW/OB group showed no significant differences between the two groups. Notably, total body mass, LM, and FM were all found to be significantly different between all groups (*p* < 0.0001 for all groups). As expected, LMI and FMI followed a similar pattern (significantly increasing, p<0.001) as BMI with a more pronounced difference in FMI across the BMI percentiles. Systolic and diastolic blood pressure were significantly different between groups (*p* < 0.001) with the SO group displaying a slightly clinically elevated systolic pressure (122 ± 13 mmHg, ~91^st^ percentile).

**Table 1:** Subject demographics and anthropometric characteristics by body mass index percentiles.

Characteristic	Normal (n = 77)	Overweight/obesity (n = 60)	Severe obesity (n = 50)	p-value
Age (years)	12.7 (2.6)	12.6 (2.5)	12.5 (2.60	0.182
Race (n[%])				0.051
White	70 (89.7)	48(80.0)	36(70.0) †	
Black	3 (3.8)	4(6.7)	4(8.0)	
American Indian	0 (0)	0(0)	1(2.0)	
Asian/Pacific Islander	1 (1.3)	3(5.0)	1(2.0)	
Other/Mixed	4 (5.1)	5(8.3)	8(16.0)	
Tanner stage [n(%)]				0.060
I	27 (35.5)	12(20.3)	4(8.2)	
II	13 (17.1)	11(18.6)	14(28.6)	
III	11 (14.5)	15(25.4)	10(20.4)	
IV	16 (21.1)	12(20.3)	12(24.5)	
V	9 (11.8)	9(15.3)	9(18.4)	
Sex (n[%])				0.114
Male	45 (57.8)	27(45.0)	20(40.0)	
Female	33 (42.3)	33(55.0)	30(60.0)	
Body mass (kg)	45.3 (12.3)	66.7 (12.9) †	96.7 (23.3) †‡	<0.0001^*^
Height (cm)	154.3 (15.1)	157.0 (13.7)	162.0 (10.7) †	0.009^*^
BMI (%tile)	48.1 (22.1)	95.0 (4.3) †	99.0 (0.4) †‡	<0.0001^*^
LMI (kg/m^2^)	13.2 (2.5)	15.6 (2.2) †	17.9 (3.8) †‡	<0.001^*^
FMI (kg/m^2^)	6.8 (2.6)	15.8 (4.8) †	26.9 (7.8) †‡	<0.001^*^
Lean mass (kg)	32.9 (10.5)	39.3 (10.8) †	48.7 (12.6) †‡	<0.0001^*^
Fat mass (kg)	10.8 (9.18)	25.1 (8.8) †	44.7 (12.5) †‡	<0.0001^*^
SBP (mmHg)	106 (10)	114 (12) †	122 (13) †‡	<0.0001^*^
DBP (mmHg)	57 (9)	59 (9)	63 (7) †‡	<0.0001^*^

BMI, body mass index; %tile, percentile; LMI, lean mass index; FMI, fat mass index; SBP, Systolic blood pressure; DBP, diastolic blood pressure.

Obesity status is presented in three categories: normal weight (e.g., 5^th^ to 85^th^ BMI-percentile), obese (e.g., 85^th^ to 95^th^ BMI-percentile, 100-120% of 95^th^ BMI-percentile), and severe obese (e.g., ≥120% of 95^th^ BMI-percentile).

Data presented as mean (standard deviation) unless noted.

^*^ Indicates significant difference between groups as determined by Analysis of Variance (*p<*0.05).

†Indicates significantly different compared to normal weight as determined by Tukey *post-hoc* pairwise comparisons (*p* < 0.05).

‡Indicates significantly different compared to obese as determined by Tukey *post-hoc* pairwise comparisons (*p* < 0.05).

### Vascular structure and function

Vascular structure and function parameters are displayed in **Table 2**. Abdominal aorta diameters (aAD) were significantly larger in the SO group compared to OW/OB and NW groups (*p* < 0.001). Both aDD and aCSD were significantly elevated between the SO and NW (*p* = 0.033 and 0.030 respectively) while abdominal aorta compliance was not significantly different between BMI stratified groups (aDC, p=0.063; aCSC, *p* = 0.869). Abdominal aorta IEM (aIEM) was significantly greater in the SO group compared to OW/OB and NW groups (*p* < 0.001). Structurally, aIMT was significantly larger in NW than OW/OB and SO groups as well as between the OW/OB and SO groups (*p* = 0.02).

**Table 2:** Abdominal aorta and carotid artery vascular parameters by obesity status as determined by body mass index percentiles.

Variable	Normal weight (n=77)	Overweight/Obesity (n=60)	Severe obesity (n=50)	*p*-value
aAD (mm)	8.97 (0.31)	9.58 (0.27)	11.05 (0.44) ^†‡^	<0.0001
aDD (%)	16.63 (0.63)	15.96 (0.68)	14.17 (0.64) ^†^	0.033
aDC(mm/mmHg)	0.028 (0.001)	0.027 (0.001)	0.024 (0.002)	0.06
aCSD (%)	36.39 (1.49)	35.26 (1.59)	30.61 (1.48) ^†^	0.03
aCSC (mm^2^/mmHg)	0.44 (0.02)	0.44 (0.03)	0.42 (0.03)	0.87
aIEM (mmHg)	846.0 (40.9)	960.4 (48.6)	1153.0 (70.8) ^†‡^	<0.001
aIMT (mm)	0.59 (0.09)	0.51 (0.17) ^†^	0.43 (0.17) ^†‡^	0.02
cAD (mm)	5.86 (0.50)	6.02 (0.07)	6.27 (0.11) ^†‡^	<0.001
cDD (%)	13.70 (2.77)	14.35 (3.10)	13.77 (3.12)	0.40
cDC (mm/mmHg)	002 (0.01)	0.01 (0.001)	0.01 (0.001) ^†^	0.15
cCSD (%)	29.40 (6.33)	30.89 (7.11)	29.55 (7.20)	0.40
cCSC (mm^2^/mmHg)	0.15 (0.05)	0.15 (0.04)	0.14 (0.04)	0.89
cIEM (mmHg)	998.91 (313.19)	1046.13 (317.40)	1191.07 (383.09) ^†^	0.005
cIMT (mm)	0.54 (0.05)	0.51 (0.07) ^†^	0.48 (0.11) ^†‡^	<0.001

Data are presented as mean (standard deviation).

Obesity status is presented in three categories: normal weight (e.g., 5^th^ to 85^th^ BMI-percentile), obese (e.g., 85^th^ to 95^th^ BMI-percentile, 100-120% of 95^th^ BMI-percentile), and severe obese (e.g., ≥120% of 95^th^ BMI-percentile).

†Indicates significantly different compared to normal weight as determined by Tukey *post-hoc* pairwise comparisons (*p* < 0.05).

‡Indicates significantly different compared to obese as determined by Tukey *post-hoc* pairwise comparisons (*p* < 0.05).

c, carotid artery; a, abdominal aorta; AD, arterial diameter; IMT, intima-media thickness; diameter distensibility and compliance (DD and DC, respectively), cross-sectional distensibility and compliance (CSD and CSC respectively), IEM, incremental elastic modulus; mm, millimetres; mm^2^/mmHg, millimeters squared per millimeters of mercury; mmHg, millimeters of mercury.

Similar to the abdominal aorta, carotid artery diameter (cAD) was also larger in the SO group than the OW/OB and NW groups (*p* < 0.001). The carotid artery displayed no significant differences in cDD, cDC, cDC, and cCSC (p = 0.40, 0.15, 0.40 and 0.89, respectively) between the BMI stratified groups cIEM were significantly greater (*p* = 0.005) in the SO compared to the OW/OB and NW groups. Similar to the abdominal aorta, cIMT was significantly larger in NW than OW/OB, and SO groups as well as between the OW/OB and SO groups (*p* < 0.001).

### Associations between body composition and vascular structure and function

#### Lean mass

Linear regression results relating vascular measures of structure and function to absolute tissue LM are displayed in **Table 3**. LM was significantly associated with an increase of aAD (0.113 mm per kg_LM,_ p<0.001, R^2^ = 0.41), aCSC (5.89 x 10^-3^mm^2^/mmHg per kg_LM_, p<0.001, R^2^ = 0.08) as well as aIEM (10.19 mmHg per kg_LM_, *p* < 0.01, R^2^ = 0.09). Similarly in the carotid artery, LM was significantly associated with an increase in cAD (0.020 mm per kg_LM_, p < 0.001, R^2^ = 0.15) and cIEM (8.68 mm per mmHg, *p* = 0.001, R^2^ = 0.08) as well as a decrease in cIMT (-1.56 x 10^-3^mm per kg_LM_, *p* = 0.01, R^2^ = 0.08). However, in the carotid artery, no relationship was observed between LM and cCSC. When adjusted for FDR, all variables remained significant.

**Table 3:** Multiple linear regression models to examine associations of aortic and carotid vascular measures with lean mass, adjusting for tanner stage, race, and sex.

Variable	Estimate (B) (95% CI)	*p*-value (R^2^)
aAD (mm)	0.113 (0.077, 0.150)	<0.001 (0.41)
aDD (%)	-0.020 (-0.103, 0.062)	0.62
aCSD (%)	-0.043 (-0.237, 0.151)	0.67
aDC (mm/mmHg)	6.419 x 10^-5^ (-1.019 x 10^-4^, 2.303 x 10^-4^)	0.45
aCSC (mm^2^/mmHg)	5.89 x 10^-3^ (2.660 x 10^-3^, 9.119 x 10^-3^)	<0.001 (0.08)
aIEM (mmHg)	10.188 (2.350, 18.026)	0.01 (0.09)
aIMT (mm)	-4.106 x 10^-3^ (-10.337 x 10^-3^, 2.126 x 10^-3^)	0.19
cAD (mm)	0.020 (0.012, 0.028)	<0.001 (0.15)
cDD (%)	-0.009 (-0.056, 0.039)	0.73
cCSD (%)	-0.019 (-0.128, 0.910)	0.74
cDC (mm/mmHg)	-2.583 x 10^-5^ (-9.217 x 10^-5^, 4.051 x 10^-5^)	0.44
cCSC (mm^2^/mmHg)	1.125 x 10^-4^ (-5.258 x 10^-4^, 7.509 x 10^-4^)	0.73
cIEM (mmHg)	8.676 (3.372, 13.981)	0.001 (0.08)
cIMT (mm)	-1.558 x 10^-3^ (-2.795 x 10^-3^, -3.205 x 10^-4^)	0.01 (0.08)

Data are presented as mean (95% confidence interval).

*p*-values <0.05 denotes significant association.

c, arotid artery; a, abdominal aorta; AD, arterial diameter; IMT, intima-media thickness; diameter distensibility and compliance (DD and DC), respectively), cross-sectional distensibility and compliance (CSD and CSC respectively); IEM, incremental elastic modulus; mm, millimetres; mm^2^/mmHg, millimeters squared per millimeters of mercury; mmHg, millimeters of mercury.

#### Lean muscle index

Linear regression results related to LMI are displayed in **Table 4**. In the abdominal aorta, LMI was significantly associated with an increase in aAD (0.41 mm per kg/m^2LMI^, *p* < 0.001, R^2^ = 0.38), aCSC (14.41 x 10^-3^mm^2^/mmHg per kg_LM_, p<0.001, R^2^ = 0.04) and aIEM (37.53 mmHg per kg/m^2LMI^, *p* < 0.01, R^2^ = 0.09) and a decrease in aIMT (-0.024 mm/mmHg per kg/m^2LMI^, *p* = 0.03, R^2^ = 0.24). Similarly in the carotid artery, LMI was also significantly associated with an increase in cAD (0.05 mm per kg/m^2LMI^, p<0.001, R^2^ = 0.09), cIEM (29.23 mmHg per kg/m^2^, *p* < 0.01, R^2^ = 0.07) and a decrease in cIMT (-7.358 x 10^-3^mm per kg/m^2LMI^, *p* < 0.001, R^2^ = 0.11). When adjusted for multiple comparisons, aIMT became statistically nonsignificant.

**Table 4:** Multiple linear regression models to examine associations of aortic and carotid vascular measures with lean mass index, adjusting for tanner stage, race, and sex.

Variable	Estimate (B) (95% CI)	*p*-value (R^2^)
aAD (mm)	0.414 (0.262, 0.565)	<0.001 (0.38)
aDD (%)	-0.092 (-0.383, 0.199)	0.53
aCSD (%)	-0.192 (-0.875, 0.492)	0.58
aDC (mm/mmHg)	7.121 x 10^-5^ (-5.153 x 10^-4^, 6.577 x 10^-4^)	0.81
aCSC (mm^2^/mmHg)	14.406 x 10^-3^ (2.792 x 10^-3^, 26.019 x 10^-3^)	0.02 (0.04)
aIEM (mmHg)	37.529 (9.777, 65.281)	<0.01 (0.09)
aIMT (mm)	-24.490 x 10^-3^ (-46.247 x 10^-3^, -2.735 x 10^-3^)	0.03*
cAD (mm)	0.050 (0.021, 0.079)	<0.001 (0.09)
cDD (%)	-0.048 (-0.217, 0.121)	0.57
cCSD (%)	-0.107 (-0.494, 0.280)	0.59
cDC (mm/mmHg)	-1.959 x 10^-3^ (-4.299 x 10^-4^, 3.803 x 10^-5^)	0.10
cCSC (mm^2^/mmHg)	-7.415 x 10^-4^ (-3.008 x 10^-4^, 1.525 x 10^-3^)	0.52
cIEM (mmHg)	29.232 (10.393, 48.072)	<0.01 (0.07)
cIMT (mm)	-7.358 x 10^-3^ (-11.670 x 10^-3^, -3.045 x 10^-3^)	<0.001 (0.11)

Data are presented as mean (95% confidence interval).

*p*-values <0.05 denotes significant association.

*Indicates loss of significant association upon Benjamini-Hochberg (BH) adjustment.

LMI, Lean mass index; c, carotid artery; a, abdominal aorta; AD, arterial diameter; IMT, intima-media thickness; diameter distensibility and compliance (DD and DC, respectively), cross-sectional distensibility and compliance (CSD and CSC respectively); IEM, incremental elastic modulus; mm, millimeters; mm^2^/mmHg, millimeters squared per millimeters of mercury; mmHg, millimeters of mercury.

#### Fat mass

Linear regression results related to absolute tissue FM are displayed in **Table 5**. In the abdominal aorta, FM was shown to be significantly associated with an increase in aAD (0.056 mm per kg_FM_, *p* < 0.001, R^2^ = 0.33), and aIEM (8.501 mmHg per kg_FM_, *p* < 0.001, R^2^ = 0.11) In the carotid artery, FM was also significantly associated with an increase in cAD (0.011mm, *p* < 0.001, R^2^ = 0.11) as well as cIEM (7.731 mm per kg_FM_, *p* < 0.001, R^2^ = 0.13) along with a decrease in cDC (-6.101 x 10^-5^mm/mmHg per kg_FM_, p<0.01, R^2^ = 0.07) as well as cIMT (-1.286x10^-3^mm per kg_FM_, *p* = 0.001, R^2^ = 0.10). Similar to lean mass, when adjusted for FDR, all variables remained significant.

**Table 5:** Multiple linear regression models to examine associations of aortic and carotid vascular measures with fat mass, adjusting for tanner stage, race, and sex.

Variable	Estimate (B) (95% CI)	*p*-value (R^2^)
aAD (mm)	0.056 (0.032, 0.080)	<0.001 (0.33)
aDD (%)	-0.033 (-0.085, 0.019)	0.21
aCSD (%)	-0.073 (-0.006, 0.001)	0.24
aDC (mm/mmHg)	-7.391 x 10^-5^ (-1.784 x 10^-4^, 3.058 x 10^-5^)	0.17
aCSC (mm^2^/mmHg)	9.254 x 10^-5^ (-2.020 x 10^-3^, 2.205 x 10^-3^)	0.93
aIEM (mmHg)	8.501 (3.620, 13.382)	<0.001 (0.11)
aIMT (mm)	-2.571 x 10^-3^ (-6.741 x 10^-3^, 9.322 x 10^-4^)	0.15
cAD (mm)	0.011 (0.005, 0.016)	<0.001 (0.11)
cDD (%)	-0.017 (-0.047, 0.013)	0.25
cCSD (%)	-0.040 (-0.109, 0.029)	0.25
cDC (mm/mmHg)	-6.101 x 10^-5^ (-1.020 x 10^-4^, -1.998 x 10^-5^)	<0.01 (0.07)
cCSC (mm^2^/mmHg)	-2.769 x 10^-4^ (-6.786 x 10^-4^, 1.248 x 10^-4^)	0.18
cIEM (mmHg)	7.731 (4.476, 10.986)	<0.001 (0.13)
cIMT (mm)	-1.286 x 10^-3^ (-2.059 x 10^-3^, -5.133 x 10^-4^)	0.001 (0.10)

Data are presented as mean (95% confidence interval).

Obesity status is presented in three categories: normal weight (e.g., 5th to 85th BMI-percentile), obese (e.g., 85th to 95th BMI-percentile, 100-120% of 95th BMI-percentile), and severe obese (e.g., ≥120% of 95th BMI-percentile).

*p*-values <0.05 denotes significant association.

c, Carotid artery; a, abdominal aorta; AD, arterial diameter; IMT, intima-media thickness; diameter distensibility and compliance (DD and DC, respectively); cross-sectional distensibility and compliance (CSD and CSC respectively); IEM, incremental elastic modulus; mm, millimetres; mm^2^/mmHg, millimeters squared per millimeters of mercury; mmHg, millimeters of mercury.

#### Fat mass index

Linear regression results related to FMI are displayed in **Table 6**. In the abdominal aorta, FMI was significantly associated with an increase in aAD (0.089 mm per kg/m^2^, *p* < 0.001, R^2^ = 0.31) as well as aIEM (14.407mmHg per kg/m^2FMI^, *p* < 0.001, R^2^ = 0.12). In the carotid artery, FMI was also significantly associated with an increase in cAD (0.016mm per kg/m^2^, *p* < 0.001, R^2^ = 0.10) and cIEM (12.785 mmHg per kg/m^2FMI^, *p* < 0.001, R^2^ = 0.13) and a decrease in cIMT (-0.002 mm per kg/m^2^, *p* < 0.001, R^2^ = 0.11) as well as cDC (-1.074 x 10^-4^mm/mmHg per kg/m^2^, *p* < 0.01, R^2^ = 0.07). Upon adjustment, all significant variables passed the BH test.

**Table 6:** Multiple linear regression models to examine associations of aortic and carotid vascular measures with fat mass index, adjusting for tanner stage, race, and sex.

Variable	Estimate (B) (95% CI)	p-value (R^2^)
aAD (mm)	0.089 (0.047, 0.131)	<0.001 (0.31)
aDD (%)	-0.056 (-0.143, 0.030)	0.20
aCSD (%)	-0.122 (-0.326, 0.081)	0.24
aDC (mm/mmHg)	-1.382 x 10^-4^ (-3.127 x 10^-4^, 3.625 x 10^-5^)	0.12
aCSC (mm2/mmHg)	-2.336 x 10^-4^ (-3.769 x 10^-3^, 3.302 x 10^-3^)	0.90
aIEM (mmHg)	14.407 (6.242, 22.571)	<0.001 (0.12)
aIMT (mm)	-4.522 x 10^-3^ (-10.270 x 10^-3^, 1.225 x 10^-3^)	0.12
cAD (mm)	0.016 (0.008, 0.025)	<0.001 (0.10)
cDD (%)	-0.027 (-0.077, 0.024)	0.30
cCSD (%)	-0.062 (-0.177, 0.054)	0.29
cDC (mm/mmHg)	-1.074 x 10^-4^ (-1.762 x 10^-4^, -3.870 x 10^-5^)	<0.01 (0.07)
cCSC (mm^2^/mmHg)	-5.399 x 10^-4^ (-1.214 x 10^-3^, 1.347 x 10^-4^)	0.12
cIEM (mmHg)	12.785 (7.317, 18.253)	<0.001 (0.13)
cIMT (mm)	-2.312 x 10^-3^ (-3.588 x 10^-3^, -1.024 x 10^-3^)	<0.001 (0.11)

Data are presented as mean (95% confidence interval).

Obesity status is presented in three categories: normal weight (e.g., 5^th^ to 85^th^ BMI-percentile), obese (e.g., 85^th^ to 95^th^ BMI-percentile, 100-120% of 95th BMI-percentile), and severe obese (e.g., ≥120% of 95^th^ BMI-percentile).

p-values <0.05 denotes significant association.

LMI, Fat mass index; c, carotid artery; a, abdominal aorta; AD, arterial diameter; IMT, intima-media thickness; diameter distensibility and compliance (DD and DC, respectively); cross-sectional distensibility and compliance (CSD and CSC respectively); IEM, incremental elastic modulus; mm, millimetres; mm ^2/^mmHg, millimeters squared per millimeters of mercury; mmHg, millimeters of mercury.

#### Combined models

**Table 7** displays the results of linear regression models combining LM and FM. When controlling for FM (and previously described variables) and adjusted for FDR, LM remained positively associated with aAD (*p* < 0.001, R^2^ = 0.44), aCSC (*p* < 0.01, R^2^ = 0.07) and cAD (*p* < 0.01, R^2^ = 0.15). Conversely, in this model FM was negatively associated with aDC (*p* = 0.01, R^2^ = 0.05), aCSC (*p* < 0.01, R^2^ = 0.12) and cDC (*p* < 0.01, R^2^ = 0.08) while showing a positive association with cIEM (*p* < 0.001, R^2^ = 0.13). The combined model results for LMI and FMI are presented in **Table 8**. Controlled for FMI and adjusted for FDR, LMI remained positively associated with aAD (*p* < 0.001, R^2^ = 0.40) and aCSC (*p* < 0.001, R^2^ = 0.07) while the only association for FMI (controlled for LMI) to survive FDR was a positive association with cIEM (*p* < 0.001, R^2^ = 0.13).

**Table 7:** Multiple linear models to examine associations of aortic and carotid vascular measures with lean mass and fat mass, adjusting for tanner stage, race, and sex.

Variable	Total lean mass (kg)		Total fat mass (kg)	
	Estimate (B) (95% CI)	p-value (R^2^)	Estimate (B) (95% CI)	p-value model (R^2^)
aAD (mm)	0.094 (0.048, 0.140)	<0.001 (0.44)	0.020 (-0.009, 0.049)	0.17
aDD (%)	0.023 (-0.085, 0.131)	0.68	-0.042 (-0.110, 0.026)	0.22
aCSD (%)	0.054 (-0.200, -0.095)	0.67	-0.095 (-0.255, 0.065)	0.24
aDC (mm/mmHg)	2.383 x 10^-4^ (2.439 x 10^-5^, 4.523 x 10^-4^)	0.03*	-1.708 x 10^-4^ (-3.060 x 10^-4^, -3.57 x 10^-5^)	0.01 (0.05)
aCSC (mm^2^/mmHg)	9.899 x 10^-3^ (5.768 x 10^-3^, 14.031 x 10^-3^)	<0.001 (0.07)	-3.933 x 10^-3^ (-6.542 x 10^-3^, -1.323 x 10^-3^)	<0.01 (0.12)
aIEM (mmHg)	2.608 (-7.508, 12.724)	0.61	7.441 (1.051, 13.831)	0.02*
aIMT (mm)	-2.134 x 10^-3^ (-10.021 x 10^-3^, 5.753 x 10^-3^)	0.58	-1.838 x 10^-3^ (-6.301 x 10^-3^, 2.624 x 10^-3^)	0.41
cAD (mm)	0.016 (0.005, 0.026)	<0.01 (0.15)	0.004 (-0.002, 0.011)	0.22
cDD (%)	0.015 (-0.046, 0.078)	0.63	-0.024 (-0.063, 0.015)	0.23
cCSD (%)	0.037 (-0.105, 0.179)	0.61	-0.055 (-0.145, 0.034	0.23
cDC (mm/mmHg)	6.037 x 10^-5^ (-2.369 x 10^-5^, 1.444 x 10^-5^)	0.16	-8.538 x 10^-5^ (-1.386 x 10^-4^, -3.223 x 10^-5^)	<0.01 (0.08)
cCSC (mm^2^/mmHg)	6.681 x 10^-4^ (-1.600 x 10^-4^, 1.484 x 10^-3^)	0.11	5.440 x 10^-4^ (-1.064 x 10^-3^, -2.440 x 10^-5^)	0.04*
cIEM (mmHg)	1.470 (-5.233, 8.180)	0.67	7.138 (2.899, 11.377)	<0.001 (0.13)
cIMT (mm)	-1.340 x 10^-3^ (-2.960 x 10^-3^, 2.800 x 10^-4^)	0.10	-1.071 x 10^-3^ (-2.030 x 10^-3^, -1.103 x 10^-4^)	0.03*

Data are presented as mean (95% confidence interval). Obesity status is presented in three categories: normal weight (e.g., 5th to 85th BMI-percentile), obese (e.g., 85th to 95th BMI-percentile, 100-120% of 95th BMI-percentile), and severe obese (e.g., ≥120% of 95th BMI-percentile).*p*-values <0.05 denotes significant association.

*Indicates loss of significant association upon Benjamini-Hochberg (BH) adjustment.

c, carotid artery; a, abdominal aorta; AD, arterial diameter; IMT, intima-media thickness; diameter distensibility and compliance (DD and DC, respectively); cross-sectional distensibility and compliance (CSD and CSC respectively); IEM, incremental elastic modulus; mm, millimetres; mm 2/mmHg, millimeters squared per millimeters of mercury; mmHg, millimeters of mercury.

**Table 8:** Multiple linear models to examine associations of aortic and carotid vascular measures with LMI and FMI, adjusting for tanner stage, race, and sex.

Variable	LMI		FMI	
	Estimate (B) (95% CI)	p-value (R^2^)	Estimate (B) (95% CI)	p-value (R2)
aAD (mm)	0.331 (0.140, 0.523)	<0.001 (0.40)	0.036 (-0.015, 0.086)	0.17
aDD (%)	0.057 (-0.330, 0.443)	0.77	-0.068 (-0.184, 0.048)	0.25
aCSD (%)	0.136 (-0.771, 1.0436)	0.77	-0.149 (-0.421, 0.123)	0.28
aDC (mm^/^mmHg)	6.621 x 10^-4^ (-1.080 x 10^-4^, 1.432 x 10^-3^)	0.09	-2.688 x 10^-4^ (-4.994 x 10^-4^,-3.816 x 10^-5^)	0.02*
aCSC (mm^2/^mmHg)	26.355 x 10^-3^ (11.094 x 10^-3^, 41.576 x 10^-3^)	<0.001 (0.070)	-5.426 x 10^-3^ (-9.990 x 10^-3^, -8.619 x 10^-4^)	0.02*
aIEM (mmHg)	10.080 (-26.383, 46.543)	0.59	12.427 (1.555, 23.299)	0.03*
aIMT (mm)	-22.909 x 10^-3^ (-52.824 x 10^-3^, 7.007 x 10^-3^)	0.13	-5.981 x 10^-4^ (-8.211 x 10^-3^, 7.015 x 10^-3^)	0.87
cAD (mm)	0.026 (-0.012, 0.065)	0.18	0.011 (-0.001, 0.022)	0.07
cDD (%)	0.018 (-0.205, 0.241)	0.87	-0.030 (-0.097, 0.036)	0.37
cCSD (%)	0.049 (-0.463, 0.560)	0.85	-0.071 (-0.224, 0.082)	0.36
cDC (mm/mmHg)	6.800 x 10^-5^ (-2.364 x 10^-4^, 3.723 x 10^-4^)	0.66	-1.208 x 10^-4^ (-2.119 x 10^-4^, -2.965 x 10^-5^)	0.01*
cCSC (mm^2/^mmHg)	-7.66 x 10^-4^ (-22.192 x 10^-4^, 37.519 x 10^-4^)	0.61	-6.900 x 10^-4^ (-1.584 x 10^-3^, 2.204 x 10^-4^)	0.13
cIEM (mmHg)	2.261 (-21.957, 26.478)	0.85	12.342 (5.092, 19.593)	<0.001 (0.13)
cIMT (mm)	-4.041 x 10^-3^ (-9.700 x 10^-3^, 1.618 x 10^-3^)	0.16	-1.521 x 10^-3^ (-3.216 x 10^-3^, 1.733 x 10^-4^)	0.08

Data are presented as mean (95% confidence interval). Obesity status is presented in three categories: normal weight (e.g., 5th to 85th BMI-percentile), obese (e.g., 85th to 95th BMI-percentile, 100-120% of 95th BMI-percentile), and severe obese (e.g., ≥120% of 95th BMI-percentile).

p-values <0.05 denotes significant association.

*Indicates loss of significant association upon Benjamini-Hochberg (BH) adjustment.

c, carotid artery; a, abdominal aorta; AD, arterial diameter; IMT, intima-media thickness; diameter distensibility and compliance (DD and DC, respectively); cross-sectional distensibility and compliance (CSD and CSC respectively); IEM, incremental elastic modulus; mm, millimetres; mm 2/mmHg, millimeters squared per millimeters of mercury; mmHg, millimeters of mercury.

## Discussion

To our knowledge this is the first study to examine the relationship of body composition biomarkers and vascular structure and function of both the carotid artery and the abdominal aorta in a young cohort of varying BMI percentiles. Additionally, in order to better understand the clinical utility of commonly reported body composition markers in the context of CVD, it is important to report the association of these indices on vascular structure and function in pediatric populations.

### Body composition and CVD risk

Within this balanced cohort of children and adolescents in respect to age, sex, Tanner stage, we observed both anthropometric, body composition, and blood pressure differences along the continuum of BMI percentile. Combined, these may put the higher BMI percentile participants at risk for future CVD related events (31). As expected, we observed that increases in BMI percentile cut-offs were accompanied by increases in both LM, FM.

#### Vascular structure

As expected, aortic and carotid diameters (aAD, cAD) were larger in SO groups reflecting their increased body mass and height. However, our findings suggest that LM specifically drives AD. First, all body composition variables absolute (LM, FM) and height adjusted (LMI, FMI) were positively associated with both arterial diameters. Secondly, after cross adjusting for FM and FMI, only LM and LMI remained significantly associated with aAD. Since LMI effectively controls for height, its sustained association, not observed in the carotid, suggests this relationship in independent of growth.

Interestingly, the NW group displayed larger IMT in both arteries compared to OW/OB, and SO groups. This paradoxical finding may be explained by a lower fat mass to lean mass ratio possibly due to increased activity level, cardiorespiratory fitness, and muscle development. These physiological alterations may result in better peripheral metabolic function as well as increase in cardiac output and vascular shear, ultimately resulting in adaptive increases in cIMT, as seen in the NW group (14, 32).

Neither FM nor FMI were associated with aIMT in our sample. Although, both FM and FMI were significantly associated with decreased cIMT and this association was preserved for absolute FM even after adjusting for LM (prior to FDR correction). These results differ from previous pediatric literature which typically reports FM being associated with increased cIMT across various age ranges as well as pubertal and obesity statuses (33–36).

Similarly, while the recent literature have reported higher LM to increased cIMT (37, 38), we observed the opposite: LM and LMI were associated with decreased cIMT with no significant associations to aIMT. These differences highlight the complex nature of pediatric arterial structure. The lack of association between aIMT and body composition in any of our models are unclear. However, it may suggest that the structure of the abdominal aorta is affected by other factors such as its proximity further from the heart outflow or its relationship to visceral organ’s (i.e., pancreas, kidneys).

#### Vascular function

Regarding vascular function, the SO group displayed a significantly higher IEM in both artery beds studied, indicative of a less elastic vascular phenotype. Another interesting observation is that increased distensibility was observed in the abdominal aorta in the SO group compared to NW which was not present in the carotid artery. It is possible that the abdominal aorta may display functional differences before they are detected in the carotid artery (28).

Functionally, FM and FMI were significantly associated with increased vessel stiffness, as reflected by increased IEM in both the abdominal aorta and carotid artery. Notably, the associations LM/LMI and IEM became insignificant when adjusted for the fat components. This reinforces that adipose tissue is more potent driver of decreased vascular function in both vascular beds. Similarly, FM and FMI were also associated with a stiffer vessel reflected by decreased compliance in both arteries.

Contrastingly, LM showed a positive association with aortic compliance which was preserved when controlled for FM. Additionally, LMI was also significantly associated with increased aortic compliance suggesting these results are independent of growth. These results are similar to those reported in normal weight children (14) and support the negative effects of absolute FM and the relatively protective nature of LM (more pronounced in the abdominal aorta) on pediatric vascular function.

As alluded to above, while LM was significantly associated with increased vessel stiffness reflected by increased IEM in both vessels, this relationship was abolished when controlled for FM. Furthermore, neither LM nor LMI were related to compliance in the carotid artery yet remained positively associated with aortic compliance. These results agree with Sletner et al. (14), who concluded that while BMI-based metrics may be ambiguous, lean mass provides a relatively protective effect on the pediatric vasculature.

### Strengths

This study possess a number of key strengths. First, it represented a well-distributed sample of children and adolescents of varying BMI percentiles. Second, body composition variables were collected by dual X-ray absorptiometry which is considered the gold standard for assessing whole body composition. Third, by incorporating both absolute masses (LM, FM) as well as height indexed variables (LMI, FMI) helped to distinguish biological effects from those associated with normal growth and maturation. Finally, the use of ultrasound with state-of-the-art wall tracking software allowed us to accurately assess both structure and function in multiple vascular locations in sequence.

### Limitations

Limitations include a modest sample size which limits the statistical power to conclude definitive causal relationships between our study variables. Though this study didn’t aim to create predictive models, the R^2^ values (not reported) were small and generally explained ~4-40% of the variability in the models in our modest sample size. However, this is common in physiological research where complex and variable biological interaction, lifestyle choices, environmental exposures all play a role and these relationships may not be completely linear (39, 40). Even a small effect (low R^2^) over a long period and steep obesity trajectory can be clinically meaningful. Furthermore, a heterogenous sample and lack of inflammatory blood markers (though beyond the scope of this study) this makes it difficult to distinguish the mechanistic nature of the observed relationships. Future studies should expand on these findings larger sample sizes in each BMI category to explore possible body composition association differences between groups as well as include blood biomarkers and ratio statistics such as the metabolic load capacity model to better understand the mechanistic nature of these differences and associations in children.

## Conclusion

In general, this study found differences in vascular structure and function across BMI percentiles in children and adolescents. A stiffer vascular profile was found in participants with severe obesity and was more pronounced in the abdominal aorta than the carotid artery. It also highlighted that absolute lean and fat mass as well as these compartments relative to stature relate slightly different to both structure and function in different vascular locations. Furthermore, it reinforces the deleterious nature of fat mass and protective effect of lean mass in terms of both absolute masses and when normalized for stature., especially in the abdominal aorta, a vessel vastly underreported in the pediatric literature. These results provide further insight into body composition biomarkers which may guide pediatric clinicians and researchers to better stratify CVD risk in relation to vascular structure and function.

## Data Availability

The datasets presented in this article are not readily available because data access may be granted by corresponding author at a reasonable request. Requests to access the datasets should be directed to denge001@umn.edu.
